# Yield determinants in rice under organic and conventional production systems: Implications for selection strategies

**DOI:** 10.1371/journal.pone.0362007

**Published:** 2026-11-03

**Authors:** Sushmitharaj Dhanalakshmi Veeraraj, Karthika Rajendran

**Affiliations:** Department of Genetics and Plant breeding, VIT School of Agricultural Innovations and Advanced Learning (VAIAL), Vellore Institute of Technology (VIT), Vellore, Tamil Nadu, India; Osmania University, INDIA

## Abstract

The persistent yield gap remains a major challenge in organic agriculture despite its increasing importance for sustainable food production. The present study compared the performance of 80 diverse rice genotypes (62 landraces  and 18 modern varieties) under two contrasting production systems (organic and conventional). The experiment was conducted during the Kharif seasons of 2023 and 2024 using an alpha lattice design (8 × 10) with three replications. Agro-morphological and yield-related traits were analysed through pooled mixed-model analysis of variance (ANOVA), genetic variability parameters, correlation and path coefficient analyses, as well as principal component analysis (PCA). The results revealed significant genotypic variation and differential trait expression between the two production systems, with the relative contribution of agro-morphological traits to grain yield varying between production systems. Integrated analysis of genetic variability, correlation, path coefficient analysis, and principal component analysis identified production system-specific trait combinations associated with grain yield. Under the conventional production system, flag leaf breadth (FLB), number of productive tillers per plant (NPTP), hundred seed weight (HSW), and flag leaf length (FLL) were prominent traits, with strong positive direct effects in path analysis, whereas leaf area (LA), NPTP, HSW, plant height (PH), and days to 50% flowering (DFF) were prominent under the organic production system, also showing positive direct effects in path analysis. Genotypic best linear unbiased prediction (BLUP) estimates identified Ponmani samba (G40), Varakkal (G60), and Sengalapattu sirumani (G49) with the highest yield BLUP estimates under the organic production system, whereas Mani samba (G29), TKM1 (G80), and Bavani (G6) showed the highest yield BLUP estimates under the conventional production system. Milagu samba (G31), Polinel (G39), and Ponmani samba (G40) consistently ranked among the top-performing genotypes based on yield BLUP estimates across both production systems, indicating their potential as breeding materials for further evaluation.

## Introduction

Providing safe and nutritious food for the growing global population while simultaneously preventing environmental degradation remains a major concern in contemporary agriculture [1]. The Food and Agriculture Organization (FAO) forecasts that global food production must increase by up to 60% by 2050 relative to 2005–2007 to adequately feed 10 billion people [2,3]. Following the Green Revolution, the use of synthetic agrochemicals accelerated agricultural productivity in conventional agriculture [4]. However, the intensive and indiscriminate use of synthetic fertilizers and pesticides in conventional (high input) production systems is frequently blamed for negative environmental consequences, including soil erosion, soil and water pollution, greenhouse gas emissions and increased insect and pest resistance [5,6]. These practices also harm human health, the soil microbiome, and ultimately affect overall ecosystem biodiversity. These long-term effects necessitate a paradigm shift towards more ecologically sound and sustainable farming practices through organic agriculture.

Over the past few decades, organic agriculture has gained increasing attention as a viable alternative for sustainable food production [7]. The International Federation of Organic Agriculture Movements (IFOAM) states that organic agriculture is mainly based on the principles of health, ecology, fairness and care. It relies on nutrient recycling, organic amendments and natural processes (biological nitrogen fixation, decomposition), thereby significantly reducing the dependency on external chemical inputs [1,8–10]. The slow-release nature of organic manures enhances soil physical properties (structure, water holding capacity and drainage) and biological properties (e.g., increased microflora), thereby protecting ecological biodiversity [6,11–13]. Globally, about 50.9 million hectares (Mha), or around 1.1% of total agricultural land is currently under organic farming, managed by approximately 2.4 million farmers [14].

Despite these clear environmental and health advantages, a significant scientific debate persists regarding the scalability of organic farming. Several authors argue that without synthetic chemicals and genetically engineered crops, organic systems simply cannot meet the future food demand of the growing global population [15–17]. Concerns encompass the potential necessity of expanding organic areas into forest and natural lands, which may result in biodiversity loss and increased greenhouse gas emissions [18]. The yield gap remains an important challenge in organic agriculture, primarily due to the cultivation of common high yielding varieties (HYVs) under organic conditions [19]. Approximately 95% of the varieties currently cultivated under organic conditions were originally bred and selected under conventional (inorganic) conditions [20–22]. These conventionally bred varieties may perform inconsistently under organic conditions, partly because nutrient release from organic amendments occurs more slowly and is less synchronized with critical crop growth stages [23]. Previous yield reports suggest about 20–50% yield reduction under organic agriculture compared with conventional systems [1,24]. Therefore, the development or introduction of varieties adapted to organic production conditions is important for improving yield stability under organic production systems.

As a prime food of nearly half of the world’s population, rice (*Oryza sativa* L.) holds a significant role in ensuring food and nutritional security [25,26]. Although organic rice breeding programmes are still in their nascent stages, renewed interest has been observed in traditional landraces because of their superior nutritional and functional qualities [27,28]. Despite their comparatively lower productivity, landraces possess excellent adaptation to local conditions, harbor untapped alleles for stress tolerance and enhanced nutritional quality, including higher amounts of secondary metabolites, and they also possess a lower glycaemic index [25,29,30]. Such diversity represents an invaluable genetic resource for organic plant breeding programmes. Therefore, agro-morphological characterization of available landraces and modern cultivars under an organic production system provides fundamental information for organic crop improvement [31].

Grain yield is a complex quantitative trait, and understanding its genetic basis is challenging because it is influenced by numerous vegetative and grain characteristics such as grain weight, grain filling, and grain number [32,33]. For the development of high yielding stable varieties, understanding the extent of genetic variability and the nature of interrelationships among agro-morphological traits is a prerequisite for effective selection. In rice, variability in agronomic traits contributes to resilience, wider adaptability and greater genetic gain across diverse environments [34,35]. Correlation and path coefficient analyses provide insights into trait interrelationships and their direct and indirect effects on yield components, thereby facilitating the identification of key traits influencing productivity [36,37]. Multivariate techniques, such as principal component analysis (PCA), reduce the multidimensionality of the data and partition the total variation into several principal components, thereby allowing identification of the major contributors to overall diversity and genotype grouping [38,39].

Although several studies have evaluated genetic variability and yield-related traits in diverse rice germplasm, most have been conducted under conventional high-input production systems or under specific nutrient management practices [40–43]. Comparative evaluation of diverse rice genotypes under contrasting organic and conventional production systems remains limited, particularly for traditional rice landraces. Consequently, differences in trait expression and genotype performance between contrasting production systems remain insufficiently understood, particularly among diverse rice landraces. Such information is essential for developing production system-specific breeding strategies and identifying suitable genotypes for sustainable rice improvement. Therefore, the present study evaluated diverse rice genotypes under contrasting organic and conventional production systems to characterize genetic variability, determine production system-specific trait associations, and identify promising breeding materials through integrated phenotypic and multivariate analyses. The findings provide a framework for production system-specific trait selection in rice breeding.

## Materials and methods

### Experimental site

The field experiments were conducted over two consecutive Kharif seasons (2023 and 2024) under two contrasting production systems (organic and conventional) at the Agricultural Farm of the VIT School of Agricultural Innovations and Advanced Learning (VAIAL), Vellore Institute of Technology, Vellore, India. The organic experiment was conducted at the certified organic research farm (12°58′07″ N, 79°09′40″ E), whereas the conventional experiment was conducted in a nearby conventionally managed experimental field (12°57′54″ N, 79°10′40″ E). The two experimental fields were located approximately 2 km apart and were situated within the same agro-climatic region. The organic field had been maintained under certified organic management for eight consecutive years prior to the initiation of the experiment. Both experimental sites experienced similar environmental conditions, including rainfall (913 mm in 2023 and 1,443 mm in 2024), temperature (25–34 °C in 2023 and 21–33 °C in 2024), and relative humidity (85% in 2023 and 81% in 2024) throughout the experimental period. Before the commencement of the experiment, soil samples were collected separately from both experimental fields using a representative composite soil sampling procedure and analysed for their physicochemical properties. The soils of both experimental fields were characterized as clay loam, and detailed information on their baseline physicochemical properties is presented in Table 1.

**Table 1:** Baseline physicochemical properties of the experimental soils before crop establishment under organic and conventional production systems during the Kharif seasons of 2023 and 2024.

Soil property	Organic (2023)	Conventional (2023)	Organic (2024)	Conventional (2024)
**pH**	7.3	7.5	7.15	7.4
**EC (mS cm^−1^)**	0.56	0.74	0.9	0.26
**Organic carbon (%)**	1.83	1.81	1.85	1.61
**Available N (mg kg^−1^)**	65.6	41.93	18.95	30.87
**Available P (mg kg^−1^)**	8.84	7.4	8.09	14.82
**Available K (mg kg^−1^)**	127	215	254	169
**Available Zn (mg kg^−1^)**	0.47	0.6	1.43	2.05
**Available Fe (mg kg^−1^)**	8.26	22.2	11.64	24.8
**Texture**	Clay loam			

Soil samples were collected before crop establishment in each cropping season using representative composite sampling from the respective experimental fields. The organic experimental field had been maintained under certified organic management for eight consecutive years prior to initiation of the experiment.

### Plant materials

The study employed a panel of 80 rice genotypes, consisting of 62 traditional landraces and 18 modern varieties from Tamil Nadu. The genotypes were obtained from various sources, including Tamil Nadu Agricultural University (TNAU), Coimbatore; Agricultural College and Research Institute (AC&RI), Madurai; Rice Research Station (RRS), Tirur; Tamil Nadu Rice Research Institute (TRRI), Aduthurai; and progressive traditional rice-growing farmers from different agro-climatic regions of Tamil Nadu. The complete list of the 80 genotypes included in the study is provided in Table 2.

**Table 2:** List of rice genotypes used in the present study.

Code	Name	Code	Name	Code	Name	Codes	Name
G1	Adukkan	G21	Karuppu kavuni	G41	Poongar	G61	Vasanai seeraga samba
G2	Annai Komban	G22	Karuthukkar	G42	Ramakali	G62	Vellai kudai valzhai
G3	Annam alagi	G23	Kattuyanam	G43	Rathasali I	G63	ADT46
G4	Arcot kitchadi	G24	Kitchadi samba	G44	Rathasali II	G64	ADT51
G5	Basmati	G25	Kitchali samba	G45	Samba mosanam	G65	ADT53
G6	Bavani	G26	Kochin samba	G46	Seeraga samba	G66	ADT54
G7	Buthakaima	G27	Kuthiraivali samba	G47	Sempalai	G67	Anna (R) 4
G8	Chinna adukku nel	G28	Kuzhiyadichan	G48	Sempuli samba	G68	ASD16
G9	Chinnar	G29	Mani samba	G49	Sengalapattu sirumani	G69	ADT37
G10	Chithiraikar	G30	Mappillai samba	G50	Sivan samba	G70	CO51
G11	Edakkal	G31	Milagu samba	G51	Sivappu kavuni	G71	CO 54
G12	Garudan samba	G32	Nattu basmati	G52	Swarna malli	G72	CO55
G13	Illupaipoo samba	G33	Navara	G53	Thanga samba	G73	CO56
G14	Indhurani	G34	Nei kitchadi	G54	Thooyamalli	G74	CR1009
G15	Jasmine	G35	Ottu kitchadi	G55	Thulasivasanai seeraga samba	G75	IR 50
G16	Kadai kalluthan	G36	Ottu kitchadi II	G56	Vadakathi samba	G76	TRY1
G17	Kalluputhan	G37	Palkudai valzhai	G57	Vaigunda Red	G77	TRY5
G18	Kaluyundaiyan	G38	Pisini	G58	Vaigunda white	G78	TKM9
G19	Kamban samba	G39	Polinel	G59	Valan samba	G79	TKM13
G20	Karunkuruvai	G40	Ponmani samba	G60	Varakkal	G80	TKM15

G1 to G62 – Rice landraces; G63 to G80 – Modern rice varieties.

### Experimental design and crop management

The experiments under both production systems were laid out in an alpha lattice design (8 × 10) with three replications. Each experimental plot comprised three rows of each genotype, each measuring 3.0 m in length. A uniform spacing of 20 cm × 20 cm was maintained between rows and plants across all the plots and environments. Prior to sowing, seeds were treated with the biofertilizers *Azospirillum* and *Phosphobacteria* following the recommended protocol. The pre-germinated seeds were raised in nursery beds, and 25-day-old seedlings were transplanted simultaneously into the respective organic and conventional fields.

Under the organic production system, crop management practices were followed according to the recommendations of TNAU AgriTech Portal, where enriched farmyard manure (750 kg ha^−1^) was applied as a basal dose, followed by vermicompost (1 t ha^−1^) at 25 and 45 days after transplanting (DAT) [44]. In addition, 3% Panchagavya was applied as a foliar spray at 15-day intervals until panicle initiation. No synthetic fertilizers or chemical pesticides were applied throughout the crop growth period. Under the conventional production system, the recommended fertilizer dose of 150:50:50 kg ha^−1^ (N: P_2_O_5_: K_2_O) was applied in split applications at basal, tillering, and panicle initiation stages along with the recommended plant protection measures. In both production systems, irrigation was managed using the alternate wetting and drying (AWD) method, manual weeding was carried out twice during the crop growth period, and all other agronomic practices were followed according to the respective production system recommendations [45].

### Data collection and trait measurement

The observed variables included agro-morphological, yield-related traits, which were recorded following the Standard Evaluation System (SES) for Rice developed by the International Rice Research Institute [46]. The recorded traits were days to 50% flowering (DFF), plant height (PH), flag leaf length (FLL), flag leaf breadth (FLB), leaf area (LA), number of tillers per plant (NTPP), number of productive tillers per plant (NPTP), panicle length (PL), number of grains per panicle (NGPP), number of filled grains per panicle (NFGPP), spikelet fertility (SF), hundred seed weight (HSW), single plant yield (SPY), kernel length (KL), and kernel breadth (KB).

Leaf area (LA) was calculated using the formula LA = K × (L × W), where LA is the leaf area (cm²), L is the leaf length (cm), W is the maximum leaf width (cm), and K is the correction factor (0.75). All the biometrical observations were recorded from five randomly selected competitive plants per replication from the middle row of each plot to minimize border effects.

### Statistical analysis

The phenotypic data recorded from 80 rice genotypes evaluated under two contrasting production systems (organic and conventional) over two consecutive Kharif seasons (2023 and 2024) were analysed using RStudio version 2025.09.1 [47]. Initially, each environment was analysed separately to assess the homogeneity of error variances. After confirming homogeneity, a pooled analysis across seasons and production systems was performed by fitting a linear mixed-effects model using the *lme4* package [48], where genotype (G), season (S), and production system (P) were considered fixed effects, while replication and block nested within replication were treated as random effects. The statistical model used was:


$$\begin{array}{c} Y_{ijkl}=\mu +G_{i}+S_{j}+P_{k}+(GS{)}_{ij}+(GP{)}_{ik}+(SP{)}_{jk}+(GSP{)}_{ijk}+\varepsilon_{ijkl} \end{array}$$

where $Y_{ijkl}$ is the observed phenotypic value of the *i*th genotype in the *j*th season under the *k*th production system and *l*th replication; μis the overa*l*l mean; $G_{i}$, $S_{j}$, and $P_{k}$ are the main fixed effects of genotype, season, and production system, respectively; $(GS{)}_{ij}$, $(GP{)}_{ik}$, and $(SP{)}_{jk}$ represent the corresponding two-way interaction effects, $(GSP{)}_{ijk}$denotes the three-way interaction effect, and $\varepsilon_{ijkl}$ is the residual error.

Descriptive statistics, genotypic and phenotypic coefficients of variation (GCV and PCV), broad-sense heritability (H²), genetic advance (GA), and genetic advance as a percentage of the mean (GAM) were estimated using the *metan* package [49]. Genotypic best linear unbiased prediction (BLUP) was estimated as genotype random effects from the fitted mixed model using the *ranef ()* function in the *lme4* package separately for the organic and conventional production systems. The resulting genotypic BLUP estimates were subsequently used for correlation, path coefficient, and principal component analyses.

Pearson's correlation analysis was performed using the *metan* package [49]. Correlation matrices and significance tests were generated using the *Hmisc* package [50], and correlation heatmaps were visualized using the *corrplot* package [51]. In both correlation and path coefficient analysis, the number of tillers per plant (NTPP) and number of grains per panicle (NGPP) were excluded because they showed high collinearity with the number of productive tillers per plant (NPTP) and number of filled grains per panicle (NFGPP), respectively. The correlation analysis was rerun using the revised dataset after excluding NTPP and NGPP. Path coefficient analysis was subsequently performed [52], considering single plant yield (SPY) as the dependent variable and the remaining yield-related traits as independent variables. Principal component analysis (PCA) was carried out using the *FactoMineR* [53] and *factoextra* [54] packages.

## Results

### Evaluation of Trait expression and variability

#### Pooled analysis of variance (ANOVA).

The pooled ANOVA was conducted across two consecutive seasons and two contrasting production systems (organic and conventional) for fifteen yield-related traits, and the results are presented in Table 3. The mean sum of squares due to genotype (G) was highly significant (p < 0.001) for all recorded traits, indicating the presence of significant variation among the evaluated rice genotypes. Environmental variation was partitioned into season (S) and production system (P) effects. The season effect was highly significant for DFF, PH, HSW and NGPP, indicating significant seasonal variation in these traits. Similarly, the production system effect was highly significant for most of the traits, except PL, NPTP, and SPY.

**Table 3:** Pooled ANOVA of rice genotypes evaluated under organic and conventional production systems.

Source	Df	DFF	PH	FLL	FLB	LA	NTPP	NPTP	PL	NGPP	NFGPP	SF	HSW	SPY	KL	KB
**Genotype(G)**	79	***	***	***	***	***	***	***	***	***	***	***	***	***	***	***
**Season (S)**	1	***	***	ns	*	ns	*	*	**	***	**	*	***	ns	ns	*
**Production system (P)**	1	***	***	***	*	***	*	ns	ns	***	***	***	*	ns	***	***
**G × S**	79	***	***	***	***	***	***	***	***	***	***	***	***	***	***	***
**G × P**	79	***	***	***	***	***	***	***	***	***	***	***	***	***	***	***
**S × P**	1	**	**	***	***	***	**	**	***	ns	ns	ns	*	ns	*	ns
**G × S × P**	79	***	***	***	***	***	***	***	***	***	***	***	***	***	***	***

*** p < 0.001; ** p < 0.01; * p < 0.05; ns – non-significant; DFF- Days to 50% flowering; PH- Plant height (cm); FLL- Flag leaf length (cm); FLB- Flag leaf breadth (cm); LA- Leaf area (cm^2^); NTPP- Number of tillers per plant; NPTP- Number of productive tillers per plant; PL- Panicle length (cm); NGPP- Number of grains per panicle; NFGPP- Number of filled grains per panicle; SF- Spikelet fertility (%); HSW- Hundred seed weight (g); SPY- Single plant yield (g); KL- Kernel length (mm); KB- Kernel breadth (mm).

The G × S and G × P interaction effects were highly significant (p < 0.001) for all the recorded traits. The S × P interaction was also significant for most of the traits, except SPY, NGPP, NFGPP, KB, and SF. Furthermore, the three-way interaction among genotype, season, and production system (G × S × P) was highly significant (p < 0.001) for all the recorded traits.

#### Mean performance and Coefficient of variation (CV).

The boxplot visualization (Fig 1) showed differences in the distribution, range, and variability of the studied traits between the two production systems. Traits such as PH, SF, and NFGPP displayed a wider data distribution under the organic production system, whereas most of the remaining traits showed overlapping interquartile ranges between the two production systems.

**Fig 1:**
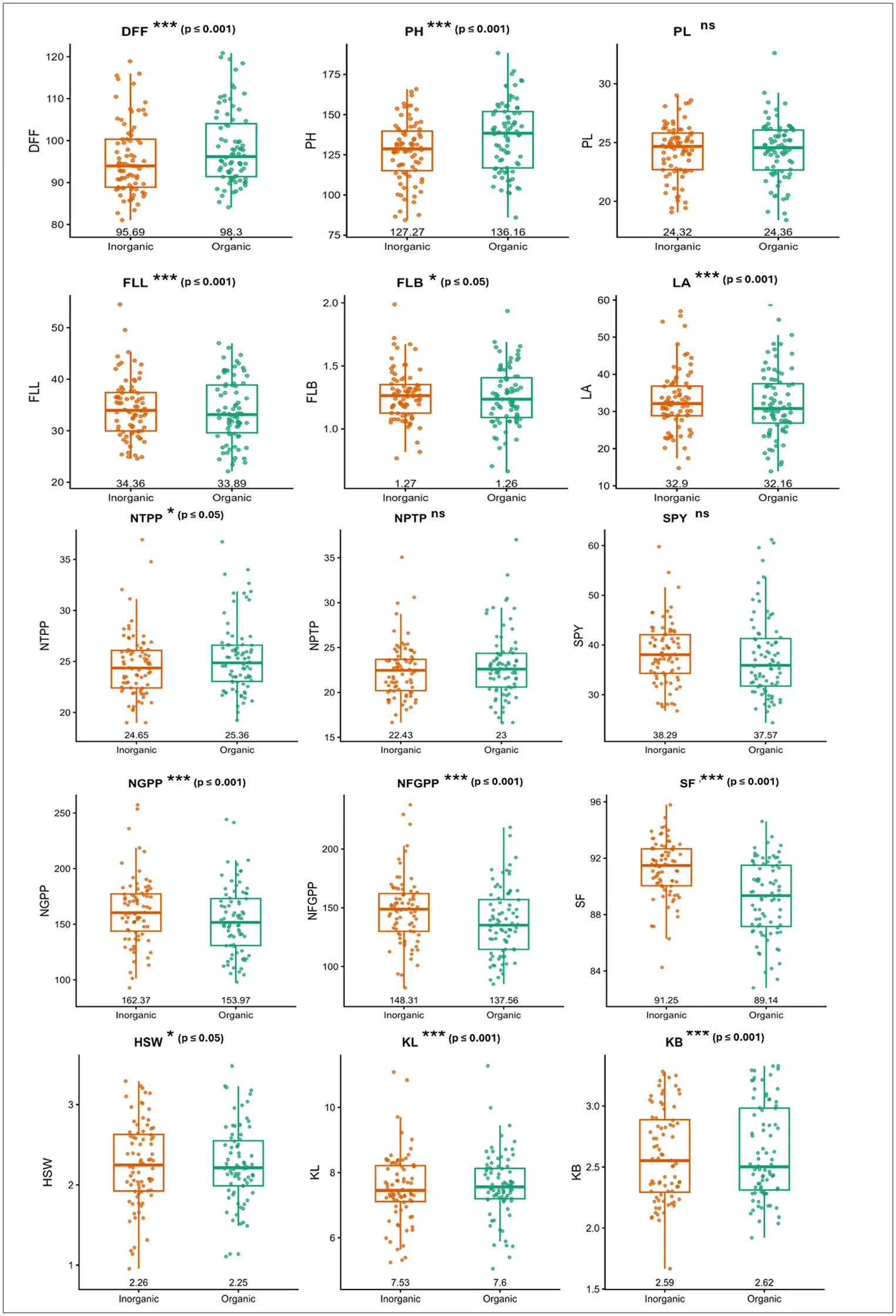
Boxplot visualization of the distribution and variation of 15 agro-morphological traits across rice genotypes under organic and conventional (inorganic) production systems. Asterisks (*) indicate significant main effects of the production system in the three-way ANOVA. DFF, Days to 50% flowering; PH, Plant height (cm); FLL, Flag leaf length (cm); FLB, Flag leaf breadth (cm); LA, Leaf area (cm2); NTPP, Number of tillers per plant; NPTP, Number of productive tillers per plant; PL, Panicle length (cm); NGPP, Number of grains per panicle; NFGPP, Number of filled grains per panicle; SF, Spikelet fertility (%); HSW, Hundred seed weight (g); SPY, Single plant yield (g); KL, Kernel length (mm); KB, Kernel breadth (mm).

The descriptive statistics presented in S1 Table showed that the mean values of PH, DFF, NTPP, and NPTP were higher under the organic production system. Plant height ranged from 80.40 cm (G71) to 192.52 cm (G40) under organic conditions, compared to 80.20 cm (G78) to 184.65 cm (G21) under conventional conditions. The mean plant height was 6.52% higher under the organic production system (p < 0.001; Fig 1).

Cultivar G68 was the earliest flowering genotype under both production systems, whereas G10 (128.74 days) and G64 (124.99 days) recorded the maximum days to 50% flowering under the organic and conventional production systems, respectively. In contrast, NGPP (−5.45%), NFGPP (−7.81%), and SPY (−2.00%) recorded lower mean values under the organic production system than under the conventional production system. The mean single plant yield was 38.42 g under the conventional production system compared to 37.68 g under the organic production system.

The coefficient of variation (CV) ranged from 3.04% (SF) to 27.59% (LA) under the conventional production system and from 4.04% (SF) to 32.50% (LA) under the organic production system. Traits such as LA, HSW, and NFGPP recorded high CV values (>20%), whereas DFF and SF exhibited low CV values (<10%) under both production systems. In addition, FLL, FLB, SPY, and NGPP exhibited high CV under the organic production system but moderate CV (10–20%) under the conventional production system.

#### Genetic variability parameters.

Estimates of phenotypic coefficient of variation (PCV) and genotypic coefficient of variation (GCV) for all the traits are presented in Table 4. The magnitude of PCV exceeded the corresponding GCV for all traits under both production systems. Under the organic production system, LA, SPY, HSW, and NFGPP recorded high PCV and GCV (> 20%), whereas under the conventional production system, only LA and HSW recorded PCV and GCV values exceeding 20%. Most of the remaining traits showed moderate variability (10–20%) at both the phenotypic and genotypic levels, while DFF and SF exhibited low variability (< 10%). The largest differences between PCV and GCV were observed for LA, NTPP, NPTP, and SPY.

**Table 4:** Genetic variability parameters, broad-sense heritability and genetic advance as percent of mean for traits evaluated under organic and conventional production systems.

Trait	Conventional production system				Organic production system			
	PCV	GCV	H^2^	GAM	PCV	GCV	H^2^	GAM
**DFF**	9.29	8.85	91	16.62	9.55	8.92	87	16.87
**PH**	15.8	14.6	85	35.37	16.76	15.71	88	41.32
**FLL**	17.94	16.49	84	10.73	20.88	17.43	70	10.15
**FLB**	17.85	15.54	76	0.35	20.66	18.08	77	0.41
**LA**	27.65	24.69	80	14.95	32.51	27.18	70	15.06
**NTPP**	16.42	11.72	51	4.25	18.37	12.28	45	4.29
**NPTP**	17.74	12.59	50	4.14	19.65	14.97	58	5.42
**PL**	10.51	9.07	75	3.92	11.35	9.87	76	4.31
**NGPP**	19.43	18.46	90	58.68	21.46	19.52	83	56.32
**NFGPP**	20.22	19.06	89	54.86	22.82	20.65	82	52.95
**SF**	3.03	2.03	45	2.55	4.05	2.72	45	3.35
**HSW**	23.57	23.11	96	1.05	22.31	21.52	93	0.96
**SPY**	19.8	15.2	63	9.24	25.36	20.94	68	13.42
**KL**	14.03	13.94	99	2.15	13.29	12.9	94	1.96
**KB**	14.82	14.38	94	0.75	15.17	14.7	94	0.77

PCV- Phenotypic coefficient of variation (%); GCV- Genotypic coefficient of variation (%); H^2^- Broad sense heritability; GAM- Genetic Advance as percent of Mean; DFF- Days to 50% flowering; PH- Plant height; FLL- Flag leaf length; FLB- Flag leaf breadth; LA- Leaf area; NTPP- Number of tillers per plant; NPTP- Number of productive tillers per plant; PL- Panicle length; NGPP- Number of grains per panicle; NFGPP- Number of filled grains per panicle; SF- Spikelet fertility; HSW- Hundred seed weight; SPY- Single plant yield; KL- Kernel length; KB- Kernel breadth (mm).

Broad-sense heritability (H²) ranged from 45 to 99% under the organic production system and from 45 to 94% under the conventional production system. Most of the evaluated traits exhibited high heritability (> 60%) under both production systems, whereas NTPP, NPTP, and SF recorded moderate heritability (30–60%). Among the evaluated traits, NGPP recorded the highest heritability, followed by NFGPP and PH under both production systems.

Genetic advance as a percentage of the mean (GAM) ranged from 0.35 to 58.68% under the organic production system and from 0.41 to 56.32% under the conventional production system. High GAM (> 20%) values were recorded for NGPP, NFGPP, and PH under both production systems, whereas DFF, FLL, LA, and SPY exhibited moderate GAM (10–20%).

### Multivariate analysis

#### Correlation and path coefficient analysis.

To examine the relationships among yield-related traits under contrasting production systems, Pearson's correlation and path coefficient analyses were performed. For path coefficient analysis, NTPP and NGPP were excluded because of their high multicollinearity with NPTP and NFGPP, respectively. The phenotypic correlation matrices are presented in Fig 2.

**Fig 2:**
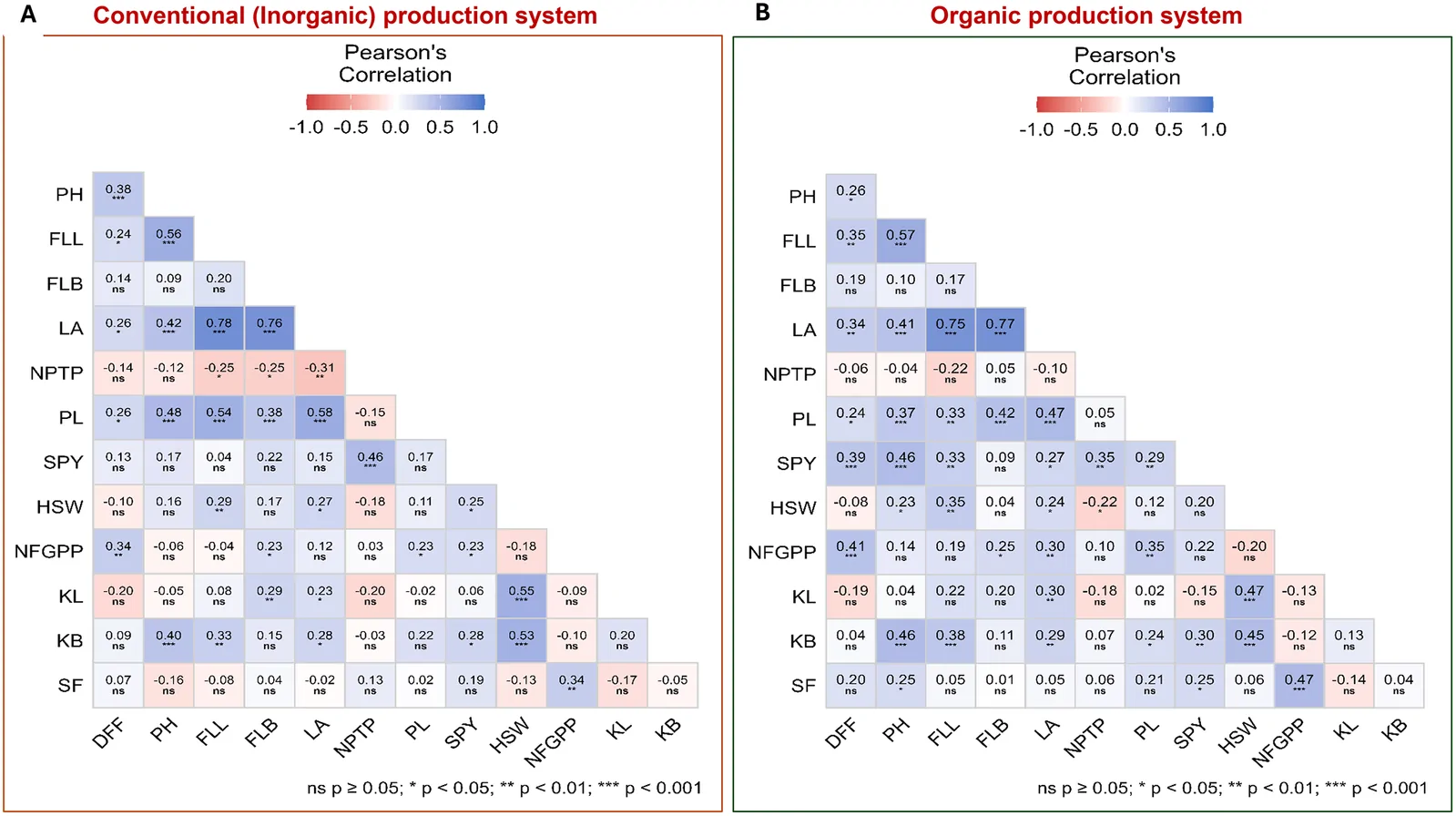
(A. B) Pearson's correlation matrices showing the relationships among agro-morphological traits of rice genotypes under conventional (A) and organic (B) production systems. DFF, Days to 50% flowering; PH, Plant height (cm); FLL, Flag leaf length (cm); FLB, Flag leaf breadth (cm); LA, Leaf area (cm?); NPTP, Number of productive tillers per plant; PL, Panicle length (cm); NFGPP, Number of filled grains per panicle; SF, Spikelet fertility (%); HSW, Hundred seed weight (g); SPY, Single plant yield (g); KL, Kernel length (mm); KB, Kernel breadth (mm).

#### Inter-trait correlations.

Plant height (PH) exhibited significant positive correlations with flag leaf length (FLL), leaf area (LA), and panicle length (PL) under both production systems. The association between PH and kernel breadth (KB) was significant only under the organic production system. Strong positive correlations were observed among leaf-related traits, particularly between FLL and LA (r = 0.78*** under conventional and 0.75*** under organic production systems, and between FLB and LA (r = 0.76*** and 0.77***, respectively). Panicle length (PL) also showed significant positive correlations with PH, FLL, FLB, and LA under both production systems, although the strength of these associations varied between production systems.

Hundred seed weight (HSW) maintained significant positive correlations with kernel length (KL) (r = 0.55*** under conventional and 0.47*** under organic production systems) and kernel breadth (KB) (r = 0.53*** and 0.45***, respectively). Number of filled grains per panicle (NFGPP) showed significant positive correlations with DFF, FLB, and PL under the conventional production system, whereas under the organic production system significant positive associations were observed with DFF, LA, and PL.

#### Single plant yield.

Under the conventional production system, single plant yield (SPY) exhibited significant positive correlations with NPTP (r = 0.46***), HSW (r = 0.25*), NFGPP (r = 0.23*), and KB (r = 0.28*). Under the organic production system, SPY showed significant positive correlations with PH (r = 0.46***), DFF (r = 0.39***), NPTP (r = 0.35**), FLL (r = 0.33**), KB (r = 0.30**), PL (r = 0.29**), and LA (r = 0.27*).

Path coefficient analysis (Table 5) revealed that under the conventional production system, FLB (0.633), NPTP (0.581), HSW (0.349), and FLL (0.317) recorded the highest positive direct effects on SPY, whereas LA showed a substantial negative direct effect (−0.603). Under the organic production system, LA (0.687), NPTP (0.422), HSW (0.332), PH (0.285), and DFF (0.283) exhibited the highest positive direct effects, while FLL (−0.356), FLB (−0.459), and KL (−0.221) recorded negative direct effects.

**Table 5:** Path coefficient analysis showing the direct and indirect effects of yield-related traits on single plant yield under conventional and organic production systems.

Traits	Conventional production system		Organic production system	
	Direct effect	Total indirect effect	Direct effect	Total indirect effect
**DFF**	0.085	0.042	0.283	0.102
**PH**	0.182	−0.011	0.285	0.172
**FLL**	0.317	−0.272	−0.356	0.683
**FLB**	0.633	−0.413	−0.459	0.553
**LA**	−0.603	0.758	0.687	−0.416
**NPTP**	0.581	−0.119	0.422	−0.074
**PL**	0.001	0.169	0.049	0.240
**NFGPP**	0.161	0.073	−0.008	0.223
**SF**	0.109	0.076	0.033	0.217
**HSW**	0.349	−0.096	0.332	−0.127
**KL**	−0.039	0.095	−0.221	0.069
**KB**	0.036	0.248	−0.023	0.322

DFF- Days to 50% flowering; PH- Plant height (cm); FLL- Flag leaf length (cm); FLB- Flag leaf breadth (cm); LA- Leaf area (cm^2^); NPTP- Number of productive tillers per plant; PL- Panicle length (cm); NFGPP- Number of filled grains per panicle; SF- Spikelet fertility (%); HSW- Hundred seed weight (g); KL- Kernel length (mm); KB- Kernel breadth (mm).

Among the indirect effects, LA exhibited contrasting patterns between the two production systems. Under the conventional production system, LA recorded the highest positive total indirect effect (0.758) despite its negative direct effect, whereas under the organic production system it exhibited the highest positive direct effect together with a negative total indirect effect (−0.416). Likewise, FLL and FLB under the organic production system recorded negative direct effects but substantial positive total indirect effects of 0.683 and 0.553, respectively. The complete matrix of indirect effects for all evaluated traits is provided in S2 Table. The residual effect of the path analysis was 0.69 under both the conventional and organic production systems.

### PCA of rice genotypes under organic and conventional (inorganic) production systems

Separate PCA was performed using fifteen quantitative agro-morphological traits of the 80 rice genotypes under conventional and organic production systems to reduce data dimensionality and summarize the major sources of phenotypic variation (Table 6). Principal components (PCs) with eigenvalues greater than one and explaining at least 5% of the total variation were retained for interpretation [55]. Of the fifteen PCs generated, the first five PCs satisfied these criteria and collectively explained 75.96% and 75.94% of the total variation under the conventional and organic production systems, respectively (Figs 3 and 4). The scree plots of eigenvalues are presented in Fig 3A and Fig 4A.

**Table 6:** Principal component analysis (PCA) summary showing eigenvalues, explained variance, cumulative variance, and trait contributions under conventional and organic production systems.

	Conventional production system					Organic production system				
**Components**	**PC1**	**PC2**	**PC3**	**PC4**	**PC5**	**PC1**	**PC2**	**PC3**	**PC4**	**PC5**
**Eigen value**	3.85	2.72	2.20	1.62	1.01	4.05	2.78	2.03	1.47	1.05
**Variance (%)**	25.69	18.11	14.66	10.78	6.71	27.01	18.56	13.52	9.83	7.03
**Cumulative (%)**	25.69	43.80	58.47	69.25	75.96	27.01	45.57	59.09	68.91	75.94
**DFF**	4.00	6.61	0.84	8.49	11.03	7.89	1.17	2.73	4.75	14.44
**PH**	9.25	0.01	3.21	18.19	3.71	9.79	2.29	2.47	10.35	0.75
**FLL**	15.15	0.44	0.85	6.93	1.66	12.08	6.97	0.01	1.12	2.81
**FLB**	10.36	0.93	0.20	10.00	20.80	7.13	0.14	0.56	31.38	1.84
**LA**	20.43	0.05	0.05	0.03	15.32	15.90	3.53	0.36	10.08	3.05
**NTPP**	6.20	5.03	23.65	0.44	2.53	0.47	14.66	22.09	4.61	0.33
**NPTP**	6.40	6.28	21.82	0.39	3.56	0.11	14.61	22.81	4.60	0.11
**PL**	12.55	2.02	0.09	3.31	3.49	10.85	0.02	0.03	1.48	0.07
**NGPP**	1.07	24.79	3.75	3.81	5.10	9.04	9.44	10.20	0.17	4.00
**NFGPP**	0.91	26.39	3.68	4.21	4.30	9.55	10.16	9.39	0.01	6.80
**SF**	0.14	8.39	0.07	1.29	0.80	3.67	3.28	0.34	10.83	28.07
**HSW**	4.97	5.30	8.00	11.74	10.61	1.29	15.37	3.92	0.55	17.08
**SPY**	0.67	7.88	20.79	2.53	0.04	8.26	1.17	11.62	5.83	1.12
**KL**	2.20	4.96	0.97	28.53	0.42	0.20	11.53	0.01	12.72	19.43
**KB**	5.71	0.92	12.04	0.13	16.64	3.78	5.69	13.47	1.53	0.08

Eigenvalues, percentage variance explained, and cumulative variance represent the contribution of each principal component to the total phenotypic variation. Values for individual traits indicate their percentage contribution to each principal component.

DFF, Days to 50% flowering; PH, Plant height (cm); FLL, Flag leaf length (cm); FLB, Flag leaf breadth (cm); LA, Leaf area (cm²); NTPP, Number of tillers per plant; NPTP, Number of productive tillers per plant; PL, Panicle length (cm); NGPP, Number of grains per panicle; NFGPP, Number of filled grains per panicle; SF, Spikelet fertility (%); HSW, Hundred seed weight (g); SPY, Single plant yield (g); KL, Kernel length (mm); KB, Kernel breadth (mm).

**Fig 3:**
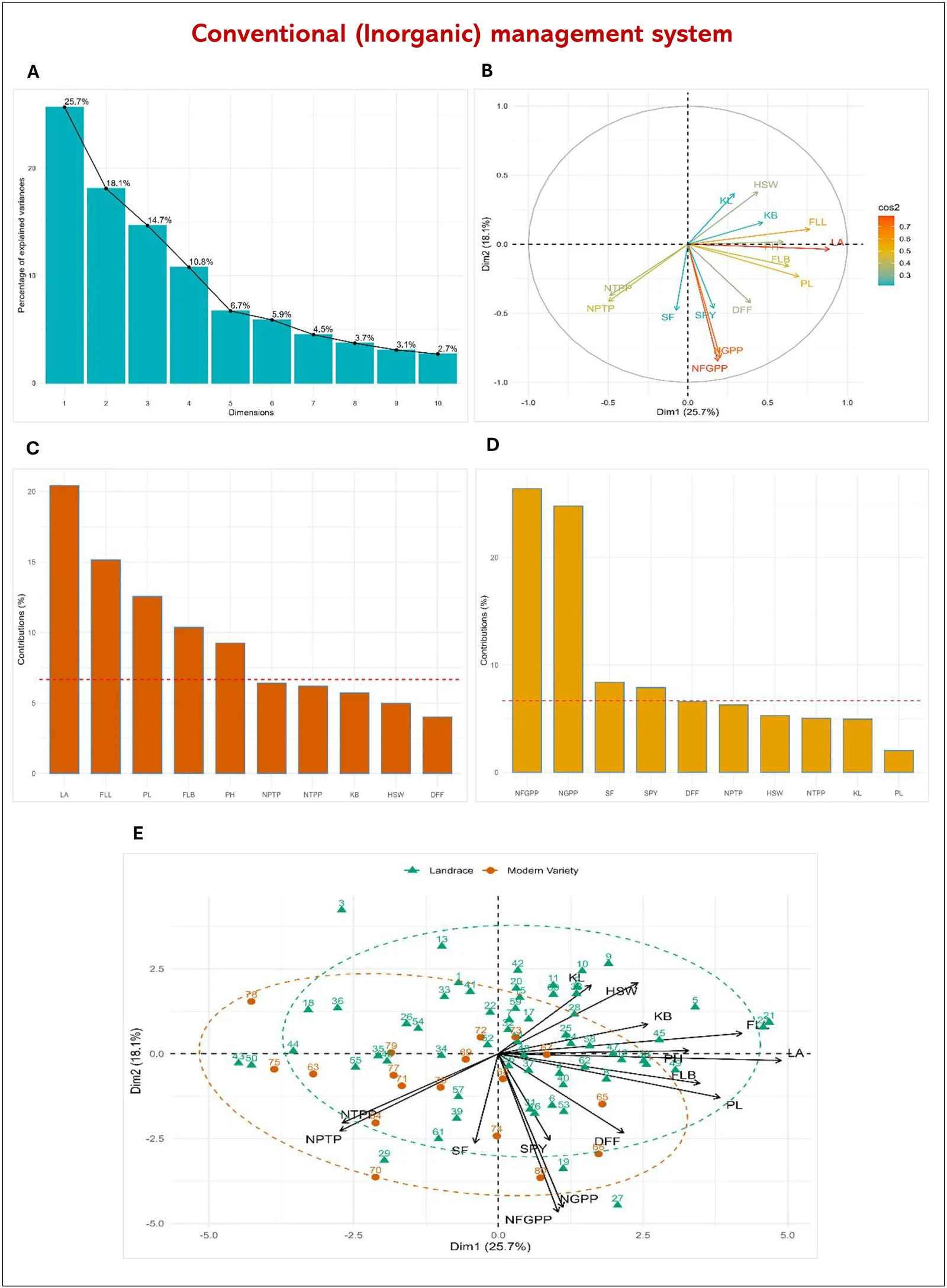
Principal component analysis (PCA) of 15 agro-morphological traits in rice genotypes under the conventional production system. **(A)** Scree plot showing the percentage of total variance explained by each principal component (PC). **(B)** PCA biplot illustrating the relationships among agro-morphological traits. **(C)** and **(D)** Bar plots showing the percentage contribution of each trait to Principal Component 1 (PC1) and Principal Component 2 (PC2), respectively. **(E)** PCA biplot showing the distribution of rice genotypes based on the first two principal components.

**Fig 4:**
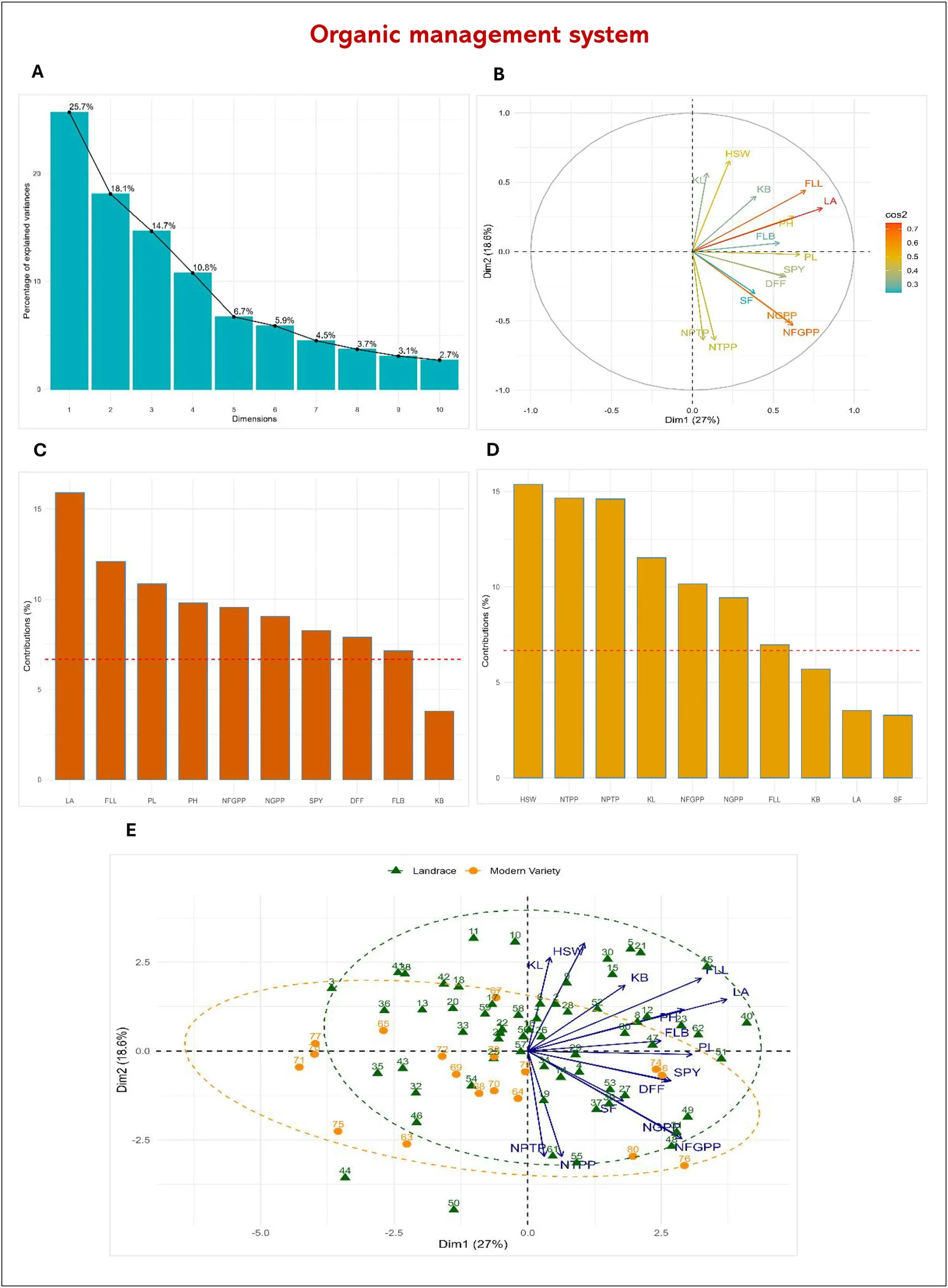
Principal component analysis (PCA) of 15 agro-morphological traits in rice genotypes under the organic production system. **(A)** Scree plot showing the percentage of total variance explained by each principal component (PC). **(B)** PCA biplot illustrating the relationships among agro-morphological traits. **(C)** and **(D)** Bar plots showing the percentage contribution of each trait to Principal Component 1 (PC1) and Principal Component 2 (PC2), respectively. **(E)** PCA biplot showing the distribution of rice genotypes based on the first two principal components.

#### Trait contributions and Interrelationships.

The contribution of individual agro-morphological traits to each principal component is presented in Table 6. Under the conventional production system (Fig 3), PC1 accounted for 25.69% of the total variation and was primarily associated with PH, FLL, LA, and PL. PC2 explained 18.11% of the variation and was mainly associated with NGPP, NFGPP, and SF. PC3 accounted for 14.66% of the variation and was characterized by NTPP, NPTP, and SPY. PC4 explained 10.77% of the variation and was mainly influenced by HSW and KL, whereas PC5 accounted for 6.71% of the variation and was associated with DFF, FLB, and KB.

Under the organic production system (Fig 4), PC1 explained 27.01% of the total variation and was primarily associated with FLL, LA, and PL. PC2 accounted for 18.56% of the variation and was mainly represented by NGPP. PC3 explained 13.52% of the variation and was associated with NTPP, NPTP, SPY, NGPP, and KB. PC4 accounted for 9.83% of the variation and was primarily associated with PH and FLB, whereas PC5 explained 7.03% of the variation and was associated with DFF, HSW, and SF.

The PCA biplots showed relatively longer vectors for LA, FLL, PH, and PL under the conventional production system, whereas FLL, PH, PL, and NFGPP displayed longer vectors under the organic production system (Figs 3D and 4D). The genotype–trait biplots (Figs 3E and 4E) illustrated the distribution of the evaluated rice genotypes with respect to the studied agro-morphological traits under both production systems. The genotypes were distributed across all four quadrants of the biplots, with varying distances from the origin. Several genotypes were positioned in the direction of individual trait vectors, indicating their association with the corresponding traits.

### Performance of genotypes based on BLUP estimates

The best linear unbiased prediction (BLUP) estimates for single plant yield (SPY) under the conventional and organic production systems are presented in Table 7. Under the organic production system, Ponmani samba (G40), Varakkal (G60), and Sengalapattu sirumani (G49) exhibited the highest genotypic BLUP estimates for SPY. Under the conventional production system, Mani samba (G29), TKM15 (G80), and Bavani (G6) exhibited the highest genotypic BLUP estimates.

**Table 7:** Top 10 rice genotypes ranked based on genotypic BLUP estimates for single plant yield (SPY) under conventional and organic production systems, together with their corresponding pooled mean SPY values.

Conventional production system				Organic production system			
**Rank**	**Genotype**	**Name**	**Mean SPY** **(g plant^−1^)**	**Rank**	**Genotype**	**Name**	**Mean SPY** **(g plant^−1^)**
1	G29	Mani samba	59.78	1	G40	Ponmani samba	61.19
2	G80	TKM15	54.59	2	G60	Varakkal	60.49
3	G6	Bavani	51.62	3	G49	Sengalapattu sirumani	59.82
4	G39	Polinel	47.60	4	G31	Milagu samba	57.00
5	G31	Milagu samba	46.82	5	G30	Mappillai samba	53.63
6	G32	Nattu basmati	46.57	6	G39	Polinel	52.51
7	G40	Ponmani samba	46.48	7	G37	Palkudai valzhai	50.26
8	G8	Chinna adukku nel	45.72	8	G23	Kattuyanam	49.22
9	G7	Buthakaima	45.64	9	G62	Vellai kudai valzhai	48.74
10	G27	Kuthiraivali samba	44.34	10	G54	Thooyamalli	46.55

Genotypes were ranked based on genotypic Best Linear Unbiased Prediction (BLUP) estimates for single plant yield (SPY). The values presented in the table represent the corresponding pooled mean SPY (g plant^−1^) of the ranked genotypes.

Among the evaluated genotypes, Milagu samba (G31), Polinel (G39), and Ponmani samba (G40) consistently ranked among the top-performing genotypes under both production systems based on their BLUP estimates. Their positions in the PCA biplots provided complementary information on their associations with the multivariate trait patterns under the two production systems.

## Discussion

### Interpreting variability and environmental influence

Grain yield in rice is a complex quantitative trait governed by multiple genes and is strongly influenced by environmental conditions throughout crop growth and development [56,57]. The highly significant genotypic effect (G) observed for all the evaluated traits indicates the presence of substantial genetic variability within the studied rice germplasm, providing considerable opportunities for effective selection and genetic improvement. Similar observations have been reported in previous studies, highlighting that genetically diverse germplasm serves as an important resource for identifying superior breeding materials [35,58–60].

The significant seasonal effects (S) observed for DFF, PH, HSW, and NGPP indicate that the expression of these traits was comparatively more responsive to seasonal environmental variation than the remaining evaluated traits [61,62]. Likewise, the significant production system effects (P) observed for most of the studied traits indicate differences in agro-morphological trait expression between the contrasting production systems [63]. Since the organic and conventional experiments were conducted in separate experimental fields with differences in baseline soil physicochemical properties, these differences should be interpreted as the combined influence of production system and experimental environment rather than production system alone.

The significant G × S and G × P interactions further indicate that the performance of rice genotypes varied across seasons and the contrasting production environments, emphasizing the importance of evaluating breeding materials under multiple production environments before selection. Furthermore, the significant three-way interaction among G × S × P highlights the complexity of trait expression under contrasting production systems and supports the identification of genotypes with stable performance under specific production environments [64–67].

### Comparative trait expression under contrasting production systems

The descriptive statistics further highlighted differences in agro-morphological trait expression between the organic and conventional production systems. The higher mean plant height and delayed flowering observed under the organic production system are consistent with previous reports describing similar responses under organic production conditions, which have been associated with differences in nutrient availability and release patterns under organic production conditions that may prolong vegetative growth [67–69]. In contrast, the higher mean values of number of grains per panicle (NGPP) and number of filled grains per panicle (NFGPP) observed under the conventional production system are in agreement with earlier studies reporting improved grain development under conditions of greater mineral nutrient availability associated with conventional fertilization [70,71]

In contrast to these traits, relatively small differences were observed for hundred seed weight (HSW), panicle length (PL), number of tillers per plant (NTPP), and number of productive tillers per plant (NPTP) between the two production systems, indicating relatively small differences in the expression of these traits between the two production systems evaluated in the present study.

Although differences in the expression of several agro-morphological traits were evident, the relatively small difference observed for single plant yield (SPY) suggests that the overall mean yield performance differed only modestly between the two production systems. In contrast, previous studies have generally reported higher grain yield under conventional production systems because of greater nutrient availability [7,63,71–73]. However, in the present study, differences in SPY between the two production systems were comparatively small, whereas several yield components showed more pronounced differences between the systems.

The higher coefficient of variation observed for several traits under the organic production system indicates greater phenotypic variability among the evaluated genotypes under these conditions. In addition, the inclusion of genetically diverse traditional landraces together with modern varieties provided a broad range of phenotypic variation for evaluating genotype performance under contrasting production systems. Together, these observations demonstrate that substantial phenotypic diversity was present across the evaluated rice germplasm under both production systems.

### Genetic variability and implications for selection

The genetic variability analysis revealed substantial genetic variation among the evaluated rice genotypes, indicating considerable scope for genetic improvement through selection [35,39]. The consistently higher phenotypic coefficient of variation (PCV) than genotypic coefficient of variation (GCV) observed for all evaluated traits indicates that both genetic and environmental factors contributed to phenotypic variation. However, the relatively small differences between PCV and GCV for most traits suggest a comparatively smaller environmental contribution to their phenotypic variation, indicating greater scope for phenotypic selection for these traits [59,61,74,75]. In contrast, the comparatively larger differences observed for leaf area (LA), number of tillers per plant (NTPP), number of productive tillers per plant (NPTP), and single plant yield (SPY) indicate a relatively greater influence of environmental factors on the expression of these traits.

Broad-sense heritability estimates provide an indication of the proportion of phenotypic variation attributable to genetic factors, whereas genetic advance as a percentage of the mean (GAM) reflects the expected improvement through selection [76]. In the present study, high heritability estimates were observed for DFF, PH, FLL, FLB, LA, PL, NGPP, NFGPP, HSW, SPY, KL, and KB under both production systems, although the magnitude varied between systems. The highest heritability estimates were observed for KL, HSW, and KB, whereas NTPP, NPTP, and SF showed moderate heritability. These results indicate substantial genetic contribution to the phenotypic variation of most evaluated traits and are consistent with earlier studies in rice under different production conditions [39,77,78].

Among the evaluated traits, PH, NGPP, and NFGPP exhibited the desirable combination of high heritability and high GAM under both production systems. PH recorded heritability of 85% and 88%, with GAM values of 35.37% and 41.32% under conventional and organic production systems, respectively. Similarly, NGPP and NFGPP recorded high heritability together with high GAM under both systems. This combination suggests a substantial contribution of genetic effects and indicates good potential for direct phenotypic selection for these traits [79,80]. In contrast, DFF, FLL, LA, and SPY exhibited moderate GAM despite moderate to high heritability, suggesting that genetic gain from direct selection may be comparatively limited and that both additive and non-additive genetic effects may contribute to their inheritance [41,81]. Consequently, genetic improvement for these traits may require selection over successive generations to achieve greater genetic gain.

Overall, the combined interpretation of PCV, GCV, heritability, and GAM indicates that PH, NGPP, and NFGPP, which expressed high heritability together with high GAM, offer greater scope for direct phenotypic selection, whereas traits showing relatively larger differences between PCV and GCV or moderate GAM require more careful evaluation across environments during the selection process.

### Trait interrelationships and yield-associated traits under contrasting production systems

Correlation and path coefficient analyses were performed to identify the agro-morphological traits associated with single plant yield (SPY) and to distinguish the direct and indirect contributions of individual traits under contrasting production systems [52,82]. NTPP and NGPP were excluded after identifying high multicollinearity with NPTP and NFGPP, respectively, and the correlation analysis was rerun using the revised dataset. The same revised dataset was subsequently used for path coefficient analysis. Correlation analysis identifies the strength of association among traits, whereas path coefficient analysis distinguishes direct effects from indirect effects by partitioning the observed correlations into direct and indirect effects.

Under the conventional production system, SPY showed significant positive associations with NPTP, HSW, NFGPP and KB. Path coefficient analysis revealed that FLB, NPTP, HSW, and FLL exerted the highest positive direct effects on SPY, indicating that productive tillering, grain weight and flag leaf-related traits were important contributors to yield expression under the conventional production system [83]. In contrast, leaf area (LA) exhibited a strong negative direct effect despite its substantial positive indirect effects through associated traits, indicating that its positive association with SPY was largely mediated through other yield-related characters rather than through a direct contribution. Similar positive relationships between productive tillers, grain weight and grain filling traits with grain yield have been reported in earlier rice studies [82,84,85].

Under the organic production system, SPY exhibited significant positive associations with DFF, PH, FLL, LA, NPTP, PL and KB, indicating that a broader range of vegetative and reproductive traits was associated with yield expression under these conditions. Path coefficient analysis identified LA, NPTP, HSW, PH and DFF as the major traits exerting positive direct effects on SPY. In contrast, FLL and FLB exhibited negative direct effects despite their positive correlations with SPY, suggesting that their contribution to grain yield was primarily mediated through indirect effects. Similar relationships among plant height, flowering duration and leaf-related traits with grain yield have been reported in rice under different production environments [59,86–88]. Previous studies have also highlighted the contribution of flag leaf characteristics to photosynthetic activity and grain filling in rice [89–91].

The contrasting patterns observed between correlation and path coefficient analyses demonstrate that significant correlation alone does not necessarily indicate a direct contribution to grain yield [39,92]. Traits such as LA under the conventional production system and FLL and FLB under the organic production system contributed to SPY predominantly through indirect associations with other yield-related traits, highlighting the importance of path coefficient analysis in distinguishing direct effects from indirect trait relationships. The production system-specific differences in direct effects further indicate that the traits most closely associated with yield differed between the two production systems, with FLB, NPTP, HSW and FLL showing the strongest positive direct effects under conventional conditions, whereas LA, NPTP, HSW, PH and DFF showed the strongest positive direct effects under organic conditions. The complete partitioning of direct and indirect effects under both production systems is presented in S2 Table. Furthermore, the comparable residual effects (0.69) observed under both production systems indicate that, although the evaluated agro-morphological traits accounted for part of the variation in SPY, additional morphological, physiological and environmental factors not included in the present model also contributed to grain yield.

### Multivariate visualization of trait expression

Principal component analysis (PCA) complemented the univariate analyses by summarizing the overall phenotypic variation among the evaluated rice genotypes into a smaller number of principal components. In the present study, the first five principal components accounted for approximately 76% of the total phenotypic variation under both production systems, indicating that most of the observed variability could be represented by a limited number of multivariate trait combinations. Similar proportions of explained variation have been reported in previous PCA studies involving rice agro-morphological traits [35,41,60,93].

The PCA further demonstrated that phenotypic variation among the evaluated rice genotypes was associated with the combined contribution of multiple agro-morphological traits rather than a single characteristic. Although the contribution of individual traits varied among the principal components, vegetative growth traits, grain number traits, yield components, and grain characteristics collectively accounted for a major proportion of the observed variation under both production systems. Under the conventional production system, the major contributions to PC1 were from PH, FLL, LA and PL, whereas NGPP, NFGPP and SF contributed prominently to PC2. NTPP, NPTP and SPY were important contributors to PC3, while HSW and KL contributed to PC4 and DFF, FLB and KB to PC5. Under the organic production system, FLL, LA and PL were prominent in PC1, NGPP in PC2, NTPP, NPTP, SPY, NGPP and KB in PC3, PH and FLB in PC4, and DFF, HSW and SF in PC5. These findings indicate that genotype differentiation under both production systems was associated with several groups of agro-morphological traits, although their relative contributions varied among the principal components.

The genotype–trait biplots further complemented the correlation and path coefficient analyses by providing a visual representation of the relationships between genotypes and the evaluated traits. Genotypes positioned in the direction of individual trait vectors exhibited closer associations with those traits, whereas their distribution across different quadrants reflected the presence of diverse trait combinations within the evaluated germplasm. Thus, the biplots provided a visual basis for identifying genotypes associated with particular combinations of agro-morphological traits and for examining the pattern of genotype differentiation under each production system. Such multivariate visualization facilitates the interpretation of complex trait relationships that may not be readily apparent from univariate statistical analyses alone.

Overall, PCA supported the presence of substantial multivariate phenotypic diversity among the evaluated genotypes and showed that genotype differentiation was associated with multiple agro-morphological traits under both production systems. The consistency of several yield-related traits, including NPTP and SPY, among the principal components, together with the system-specific contribution of traits such as PH, LA, FLB and HSW, complements the trait associations identified through correlation and path analyses. Thus, PCA provided a comprehensive overview of the multivariate phenotypic diversity present among the evaluated rice genotypes and helped characterize the trait combinations contributing to genotype differentiation under contrasting production systems.

### Breeding implications for contrasting production systems

The present study evaluated a diverse panel of 80 rice genotypes, comprising 62 traditional landraces and 18 modern varieties collected from different agro-climatic regions of Tamil Nadu, under contrasting organic and conventional production systems. This diverse germplasm panel facilitated the identification of production system-specific trait associations and promising breeding materials under the evaluated production environments. Rather than relying on a single statistical method, the integrated analytical approach adopted in the present study provided complementary insights into genotype performance, trait expression, and the relative importance of agro-morphological traits, thereby establishing a more comprehensive framework for production system-specific trait selection in rice breeding.

The results suggest that a common selection strategy may not be equally effective across contrasting production systems because the relative contribution of agro-morphological traits to grain yield differed between the organic and conventional production systems. Under the conventional production system, FLB, NPTP, HSW and FLL showed the strongest positive direct effects on SPY, whereas under the organic production system, LA, NPTP, HSW, PH and DFF showed the strongest positive direct effects. Therefore, selection criteria should be aligned with the target production system [22,94–97]. The proposed breeding framework (Fig 5) integrates the major findings of the present study and provides a practical guide for production system-specific trait selection. Such an approach may improve breeding efficiency by enabling breeders to prioritize traits that showed stronger associations with grain yield under the intended production system rather than relying on a uniform selection strategy.

**Fig 5:**
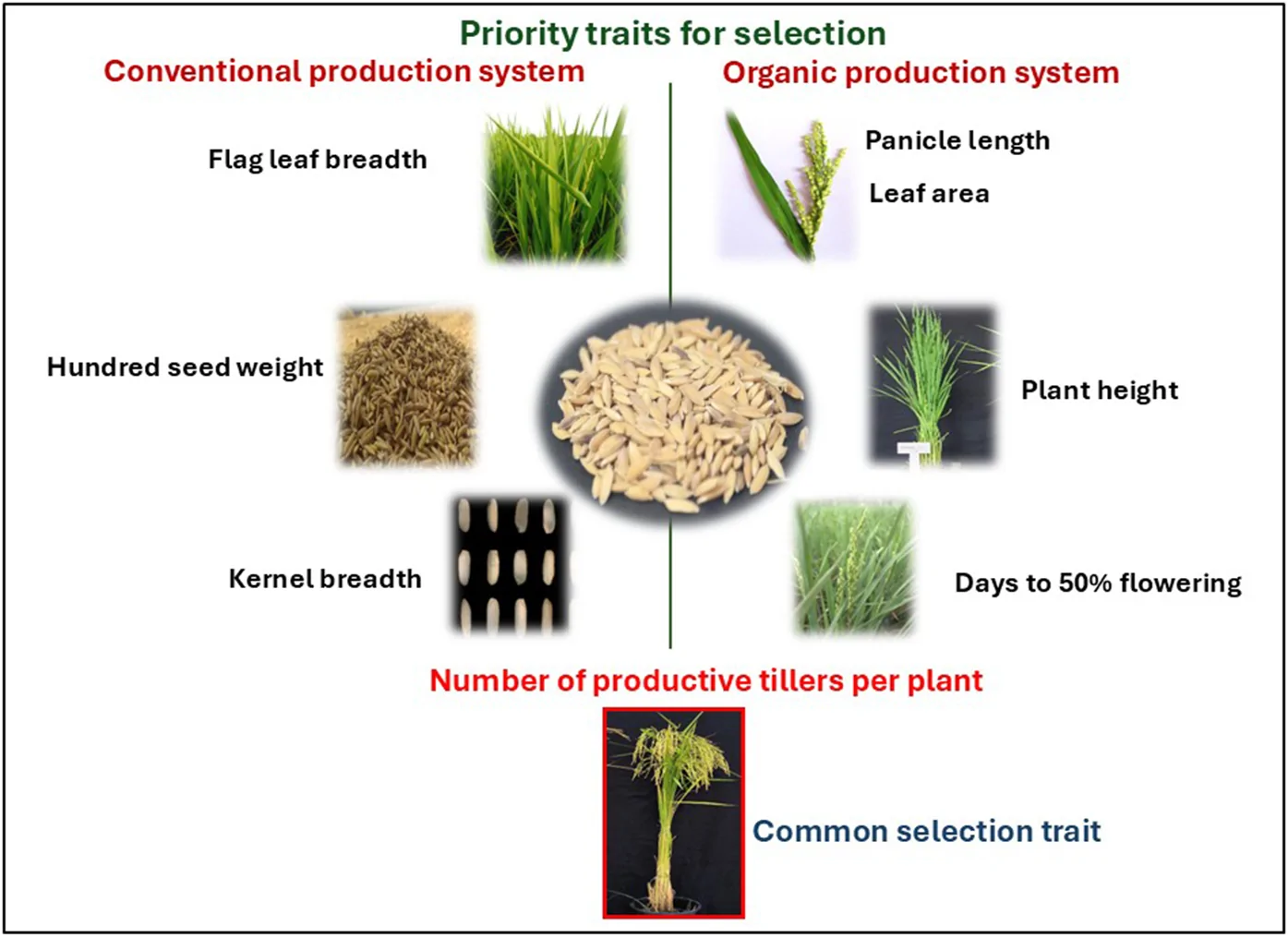
Proposed production system-specific trait selection framework for rice breeding based on the integrated interpretation of genetic variability, correlation, path coefficient analysis and principal component analysis (PCA) under conventional and organic production systems. The framework summarizes the priority agro-morphological traits associated with grain yield under each production system and highlights the common selection trait identified across both production systems to facilitate production system-specific breeding decisions.

The genotypic BLUP estimates further supported the identification of promising breeding materials for yield improvement. Ponmani samba (G40), Varakkal (G60), and Sengalapattu sirumani (G49) showed the highest SPY BLUP estimates under the organic production system, whereas Mani samba (G29), TKM13 (G80), and Bavani (G6) showed the highest SPY BLUP estimates under the conventional production system. Under organic production, G40, G60, and G49 recorded higher LA and PH values than the organic mean, while G60 and G49 also exhibited higher NPTP, and G40 and G60 recorded higher HSW, corresponding to the traits that showed strong positive direct effects on SPY under this production system. Under conventional production, G80 and G6 recorded higher FLB, NPTP, and HSW values than the conventional mean, while G29 showed higher NPTP and HSW, corresponding to traits identified through path analysis as having strong positive direct effects on SPY. The correspondence between high-yielding genotypes identified by BLUP and the traits showing strong direct effects in path analysis varied among genotypes, suggesting that high yield performance may be associated with different combinations of yield-related traits. The PCA results provided complementary evidence of multivariate trait associations and genotype differentiation under the two production systems. In addition, Milagu samba (G31), Polinel (G39), and Ponmani samba (G40) consistently ranked among the top-performing genotypes based on BLUP estimates across both production systems, indicating their potential as breeding materials for further evaluation for yield improvement. However, their performance should be further validated across multiple locations and production environments before broader utilization in varietal improvement programmes.

#### Study limitations and future perspectives.

The present study was conducted over two consecutive seasons under organic and conventional production systems established in separate experimental fields. Although the fields were located within the same agro-climatic region and experienced similar climatic conditions, differences in baseline soil physicochemical properties between the fields may also have contributed to the observed phenotypic variation. The study was primarily designed for phenotypic characterization of a diverse rice panel comprising landraces and modern varieties for yield and its contributing traits, with the objective of identifying promising genotypes and key traits for selection under organic production conditions and further breeding. Because the production systems were established as separate field environments, the baseline soil properties were not independently replicated across the production systems and therefore could not be included as covariates to separate field- and soil-specific effects from production-system effects. Therefore, differences observed between the production systems should be interpreted as reflecting the combined influence of production system and experimental environment rather than effects attributable solely to nutrient management. In addition, the study was conducted at a single location using a fixed set of rice genotypes; therefore, the observed genotype performance and trait associations require validation across diverse agro-climatic regions and production environments before broader generalization.

Future studies involving multi-location and multi-season evaluation under contrasting production systems will provide a better understanding of genotype performance and the stability of trait expression across environments. The application of stability analyses, such as additive main effects and multiplicative interaction (AMMI) and genotype plus genotype-by-environment (GGE) biplot models, together with mixed-model approaches, would facilitate the identification of genotypes with stable performance and specific adaptation across diverse environments. Subsequent agronomic evaluation of selected genotypes under different nutrient sources, application levels, and crop management practices would provide further validation of their performance and help refine trait-based selection under organic production conditions. Furthermore, integrating agro-morphological evaluation with molecular markers, genomic prediction, and other advanced genomic approaches would improve the understanding of the genetic basis of important agronomic traits and strengthen selection efficiency in rice breeding programmes. Recent advances in artificial intelligence and machine learning also provide promising opportunities for integrating large phenotypic and genomic datasets to accelerate breeding decisions and enhance genetic gain [98–100]. Overall, addressing these limitations through multi-environment validation, agronomic evaluation, and the integration of advanced phenotypic, genomic, and computational approaches will contribute to the development of more robust, efficient, and production system-specific rice breeding strategies.

## Conclusion

The present study demonstrated that the relative importance of agro-morphological traits associated with grain yield differed between the organic and conventional production systems, indicating that a common selection strategy may not be equally effective across contrasting production environments. The integrated analyses identified production system-specific trait combinations associated with grain yield, with FLB, NPTP, HSW and FLL showing strong positive direct effects under conventional production system, while LA, NPTP, HSW, PH and DFF showed strong positive direct effects under organic production system. These findings emphasize the importance of aligning trait-based selection with the target production environment to improve breeding efficiency. Among the evaluated genotypes, Milagu samba (G31), Polinel (G39) and Ponmani samba (G40) consistently ranked among the top-performing genotypes across both production systems, indicating their potential as breeding materials for further evaluation, subject to validation across diverse production environments.

## Supporting information

S1 Table Descriptive statistics of the 15 agro-morphological traits evaluated in rice genotypes under conventional and organic production systems. (DOCX)

S2 Table Indirect effects of agro-morphological traits on single plant yield estimated by path coefficient analysis under conventional and organic production systems. (DOCX)
