# Supplementary material for: Yield determinants in rice under organic and conventional production systems: Implications for selection strategies
Source: PLoS One. 2026 Nov 3;21(11):e0362007. doi: 10.1371/journal.pone.0362007 (PMC13730377; doi:10.1371/journal.pone.0362007)
Supplement: Supplementary file 1 [file pone.0362007.s001.docx]

**Supplementary Table S1. Descriptive statistics of the 15 agro-morphological traits evaluated in rice genotypes under conventional and organic production systems.**

|  | **Conventional production system** | | | | **Organic production system** | | | |
| --- | --- | --- | --- | --- | --- | --- | --- | --- |
| **Trait** | **Mean ± SE** | **Min** | **Max** | **CV** | **Mean ± SE** | **Min** | **Max** | **CV** |
| **DFF** | 95.69±0.42 | 78.49 | 124.99 | 9.72 | 98.30±0.45 | 81 | 128.74 | 10.11 |
| **PH** | 127.27±0.92 | 80.2 | 184.65 | 15.82 | 136.16±1.04 | 80.4 | 192.52 | 16.72 |
| **FLL** | 34.36±0.28 | 20.36 | 56 | 17.92 | 33.89±0.32 | 19.89 | 57.5 | 20.85 |
| **FLB** | 1.27±0.01 | 0.5 | 2.15 | 17.8 | 1.26±0.01 | 0.42 | 2.12 | 20.65 |
| **LA** | 32.90±0.41 | 9.51 | 69.4 | 27.59 | 32.16±0.48 | 8.1 | 70.28 | 32.5 |
| **NTPP** | 24.67±0.19 | 12 | 46 | 16.46 | 25.41±0.22 | 15.7 | 49 | 18.79 |
| **NPTP** | 22.50±0.18 | 11 | 43 | 17.77 | 23.06±0.21 | 12.7 | 47 | 19.98 |
| **PL** | 24.32±0.12 | 16.7 | 32.25 | 10.54 | 24.36±0.13 | 16.98 | 34.5 | 11.31 |
| **NGPP** | 162.37±1.44 | 83.96 | 281 | 19.37 | 153.97±1.50 | 88.3 | 278.12 | 21.38 |
| **NFGPP** | 148.28±1.36 | 74.14 | 261.2 | 20.15 | 137.56±1.43 | 73.7 | 236.5 | 22.72 |
| **SF** | 91.22±0.13 | 80.17 | 98.11 | 3.04 | 89.14±0.16 | 75.19 | 98.2 | 4.04 |
| **HSW** | 2.26±0.02 | 0.91 | 3.34 | 23.47 | 2.25±0.02 | 0.89 | 3.61 | 22.21 |
| **SPY** | 38.42±0.35 | 20.83 | 67.5 | 19.77 | 37.68±0.44 | 20.5 | 75.9 | 25.42 |
| **KL** | 7.53±0.05 | 5.12 | 11.2 | 13.96 | 7.60±0.05 | 4.95 | 11.86 | 13.23 |
| **KB** | 2.59±0.02 | 1.5 | 3.5 | 14.75 | 2.62±0.02 | 1.87 | 3.46 | 15.1 |

SE- Standard Error; Min- Minimum; Max- Maximum, CV- Coefficient of variation (%); DFF- Days to 50% flowering; PH- Plant height (cm); FLL- Flag leaf length (cm); FLB- Flag leaf breadth (cm); LA- Leaf area (cm^2^); NTPP- Number of tillers per plant; NPTP- Number of productive tillers per plant; PL- Panicle length (cm); NGPP- Number of grains per panicle; NFGPP- Number of filled grains per panicle; SF- Spikelet fertility (%); HSW- Hundred seed weight (g); SPY- Single plant yield (g); KL- Kernel length (mm); KB- Kernel breadth (mm).
