# Supplementary material for: Yield determinants in rice under organic and conventional production systems: Implications for selection strategies
Source: PLoS One. 2026 Nov 3;21(11):e0362007. doi: 10.1371/journal.pone.0362007 (PMC13730377; doi:10.1371/journal.pone.0362007)
Supplement: Supplementary file 2 [file pone.0362007.s002.docx]

**Supplementary Table S2. Indirect effects of agro-morphological traits on single plant yield estimated by path coefficient analysis under conventional and organic production systems.**

| **Organic production system** | | | | | | | | | | | | | | | | | | | | |
| --- | --- | --- | --- | --- | --- | --- | --- | --- | --- | --- | --- | --- | --- | --- | --- | --- | --- | --- | --- | --- |
| **predictor** | **PH** | | **FLL** | | **FLB** | | **LA** | | **PL** | | **NPTP** | | **HSW** | | **NFGPP** | **KL** | **KB** | **SF** | **DFF** |  |
| **DFF** | 0.074 | | -0.125 | | -0.087 | | 0.237 | | 0.012 | | -0.026 | | -0.026 | | -0.003 | 0.041 | -0.001 | 0.007 |  |  |
| **FLB** | 0.029 | | -0.061 | |  | | 0.526 | | 0.021 | | 0.020 | | 0.012 | | -0.002 | -0.044 | -0.003 | 0.000 | 0.054 |  |
| **FLL** | 0.162 | |  | | -0.079 | | 0.518 | | 0.016 | | -0.092 | | 0.115 | | -0.002 | -0.048 | -0.009 | 0.002 | 0.099 |  |
| **HSW** | 0.066 | | -0.123 | | -0.017 | | 0.167 | | 0.006 | | -0.094 | |  | | 0.002 | -0.103 | -0.010 | 0.002 | -0.022 |  |
| **KB** | 0.132 | | -0.135 | | -0.051 | | 0.202 | | 0.012 | | 0.029 | | 0.149 | | 0.001 | -0.028 |  | 0.001 | 0.011 |  |
| **KL** | 0.010 | | -0.078 | | -0.090 | | 0.206 | | 0.001 | | -0.075 | | 0.155 | | 0.001 |  | -0.003 | -0.004 | -0.053 |  |
| **LA** | 0.118 | | -0.269 | | -0.351 | |  | | 0.023 | | -0.042 | | 0.081 | | -0.002 | -0.066 | -0.007 | 0.002 | 0.097 |  |
| **NFGPP** | 0.041 | | -0.069 | | -0.114 | | 0.209 | | 0.017 | | 0.044 | | -0.067 | |  | 0.028 | 0.003 | 0.015 | 0.117 |  |
| **NPTP** | -0.012 | | 0.077 | | -0.021 | | -0.068 | | 0.003 | |  | | -0.074 | | -0.001 | 0.039 | -0.002 | 0.002 | -0.017 |  |
| **PH** |  | | -0.202 | | -0.047 | | 0.284 | | 0.018 | | -0.018 | | 0.077 | | -0.001 | -0.008 | -0.011 | 0.008 | 0.073 |  |
| **PL** | 0.105 | | -0.118 | | -0.192 | | 0.323 | |  | | 0.023 | | 0.039 | | -0.003 | -0.005 | -0.005 | 0.007 | 0.067 |  |
| **SF** | 0.071 | | -0.020 | | -0.005 | | 0.032 | | 0.010 | | 0.024 | | 0.021 | | -0.004 | 0.030 | -0.001 |  | 0.057 |  |
| **Conventional production system** | | | | | | | | | | | | | | | | | | | | |
| **Predictor** | | **PH** | | **FLL** | | **FLB** | | **LA** | | **PL** | | **NPTP** | | **HSW** | **NFGPP** | **KL** | **KB** | **SF** | **DFF** |  |
| **DFF** | | 0.069 | | 0.077 | | 0.092 | | -0.155 | | 0.000 | | -0.079 | | -0.035 | 0.055 | 0.008 | 0.003 | 0.007 |  |  |
| **FLB** | | 0.017 | | 0.064 | |  | | -0.457 | | 0.000 | | -0.145 | | 0.059 | 0.038 | -0.011 | 0.005 | 0.004 | 0.012 |  |
| **FLL** | | 0.103 | |  | | 0.129 | | -0.473 | | 0.001 | | -0.146 | | 0.102 | -0.007 | -0.003 | 0.012 | -0.009 | 0.021 |  |
| **HSW** | | 0.029 | | 0.092 | | 0.107 | | -0.165 | | 0.000 | | -0.106 | |  | -0.028 | -0.021 | 0.019 | -0.014 | -0.009 |  |
| **KB** | | 0.072 | | 0.104 | | 0.095 | | -0.170 | | 0.000 | | -0.015 | | 0.184 | -0.016 | -0.008 |  | -0.006 | 0.007 |  |
| **KL** | | -0.009 | | 0.026 | | 0.184 | | -0.136 | | 0.000 | | -0.119 | | 0.191 | -0.015 |  | 0.007 | -0.019 | -0.017 |  |
| **LA** | | 0.076 | | 0.248 | | 0.480 | |  | | 0.001 | | -0.183 | | 0.096 | 0.019 | -0.009 | 0.010 | -0.002 | 0.022 |  |
| **NFGPP** | | -0.011 | | -0.014 | | 0.148 | | -0.072 | | 0.000 | | 0.018 | | -0.061 |  | 0.004 | -0.004 | 0.037 | 0.029 |  |
| **NPTP** | | -0.022 | | -0.079 | | -0.158 | | 0.190 | | 0.000 | |  | | -0.063 | 0.005 | 0.008 | -0.001 | 0.014 | -0.012 |  |
| **PH** | |  | | 0.178 | | 0.058 | | -0.253 | | 0.001 | | -0.071 | | 0.055 | -0.010 | 0.002 | 0.014 | -0.017 | 0.032 |  |
| **PL** | | 0.087 | | 0.172 | | 0.238 | | -0.348 | |  | | -0.090 | | 0.039 | 0.037 | 0.001 | 0.008 | 0.002 | 0.023 |  |
| **SF** | | -0.029 | | -0.026 | | 0.025 | | 0.011 | | 0.000 | | 0.074 | | -0.044 | 0.054 | 0.007 | -0.002 |  | 0.006 |  |

Values represent the indirect effects of each predictor trait on single plant yield (SPY) through the corresponding agro-morphological traits.
